# Book Notices

**Published:** 1885-11

**Authors:** 


### BOOK NOTICES.

EPITHELIOMA OF THE MOUTH. By H. I. Ostrom, M. D. Pp. 120. New York: A. L. Chatterton, 1885.

Dr. Ostrom has acquired a favorable reputation as a successful surgeon and prescriber, therefore anything he writes on his specialty—surgery—will be found practical, sensible, and useful. It is so with this monograph on *Epithelioma of the Mouth.* To those who desire to study carefully this dread disease we can recommend this book. It is divided into two chapters—the first treats of the epithelium of the mouth, the second of epithelioma of the lip, gums, tongue, etc. We see the Doctor speaks highly of Ranunculus bulb as a remedy in these affections, but at present he finds remedies are not able to cope with it, and so operative measures have to be resorted to, which is at best only palliation.

RATIONALISM IN MEDICINE. By William Thornton. Pp. 46; price, $1.00. Boston, 1885.

Dr. Thornton, being dissatisfied with existing old school medicine, attempts in this volume to theorize concerning a rational system of medicine. To our mind, his theories are as wide of the mark as those of any of the ancients. He writes: "I have constantly heard and hear to-day of the 'science of medicine,' but have yet to learn how empiricism can become a science." There is a science of medicine, Doctor, guided by *a law of nature.* Study and investigate Homœopathy—we mean Homœopathy, not mongrelism —and you will surely be convinced there is rationalism in medicine.

Although we cannot agree with Dr. Thornton's views, we must say he presents them in a very interesting manner.

### History of Homœopathy. By Wilhelm Ameke, M. D., of Berlin, edited by Dr. R. E. Dudgeon. Pp. 445. London: E. Gould & Son, 1885.

In this interesting volume we have a full sketch of Hahnemann's life, of his works, of the reception he met with, and that Homœopathy has since met. The book is a very useful and a very interesting one, and one that laymen would do well to read carefully. Those homœopathic physicians who are not well acquainted with the early history of Homœopathy would also do well to read it.

Dr. Ameke gives us another fresh and vivid sketch of the grand old master —Samuel Hahnemann—showing clearly how wonderfully able, learned, and discriminating he was in all he did. How immeasurably greater than any of the pigmies, who in these latter days criticise and condemn him and his teachings because their little minds cannot know all he knew.

